# Cryo-EM structures of intermediates suggest an alternative catalytic reaction cycle for cytochrome *c* oxidase

**DOI:** 10.1038/s41467-021-27174-y

**Published:** 2021-11-25

**Authors:** F. Kolbe, S. Safarian, Ż. Piórek, S. Welsch, H. Müller, H. Michel

**Affiliations:** 1 Department of Molecular Membrane Biology, Max Planck Institute of Biophysics, D-60438 Frankfurt/Main, Germany; 2 Central Electron Microscopy Facility, Max Planck Institute of Biophysics, D-60438 Frankfurt am Main, Germany; 3 Present Address: Institute of Pharmaceutical Chemistry, Phillips University Marburg, D-35032 Marburg, Germany

**Keywords:** Enzyme mechanisms, Cryoelectron microscopy, Membrane proteins

## Abstract

Cytochrome *c* oxidases are among the most important and fundamental enzymes of life. Integrated into membranes they use four electrons from cytochrome *c* molecules to reduce molecular oxygen (dioxygen) to water. Their catalytic cycle has been considered to start with the oxidized form. Subsequent electron transfers lead to the **E**-state, the **R**-state (which binds oxygen), the **P**-state (with an already split dioxygen bond), the **F**-state and the **O**-state again. Here, we determined structures of up to 1.9 Å resolution of these intermediates by single particle cryo-EM. Our results suggest that in the **O**-state the active site contains a peroxide dianion and in the **P**-state possibly an intact dioxygen molecule, the **F**-state may contain a superoxide anion. Thus, the enzyme’s catalytic cycle may have to be turned by 180 degrees.

## Abstract (web summary)

Cytochrome *c* oxidase is a fundamental enzyme of life and its mechanism is not fully understood yet. Here, the authors present four cryo-EM structures of different intermediate states, which suggest an alternative cytochrome *c* oxidase reaction cycle.

## Introduction

**Fig. 1:**
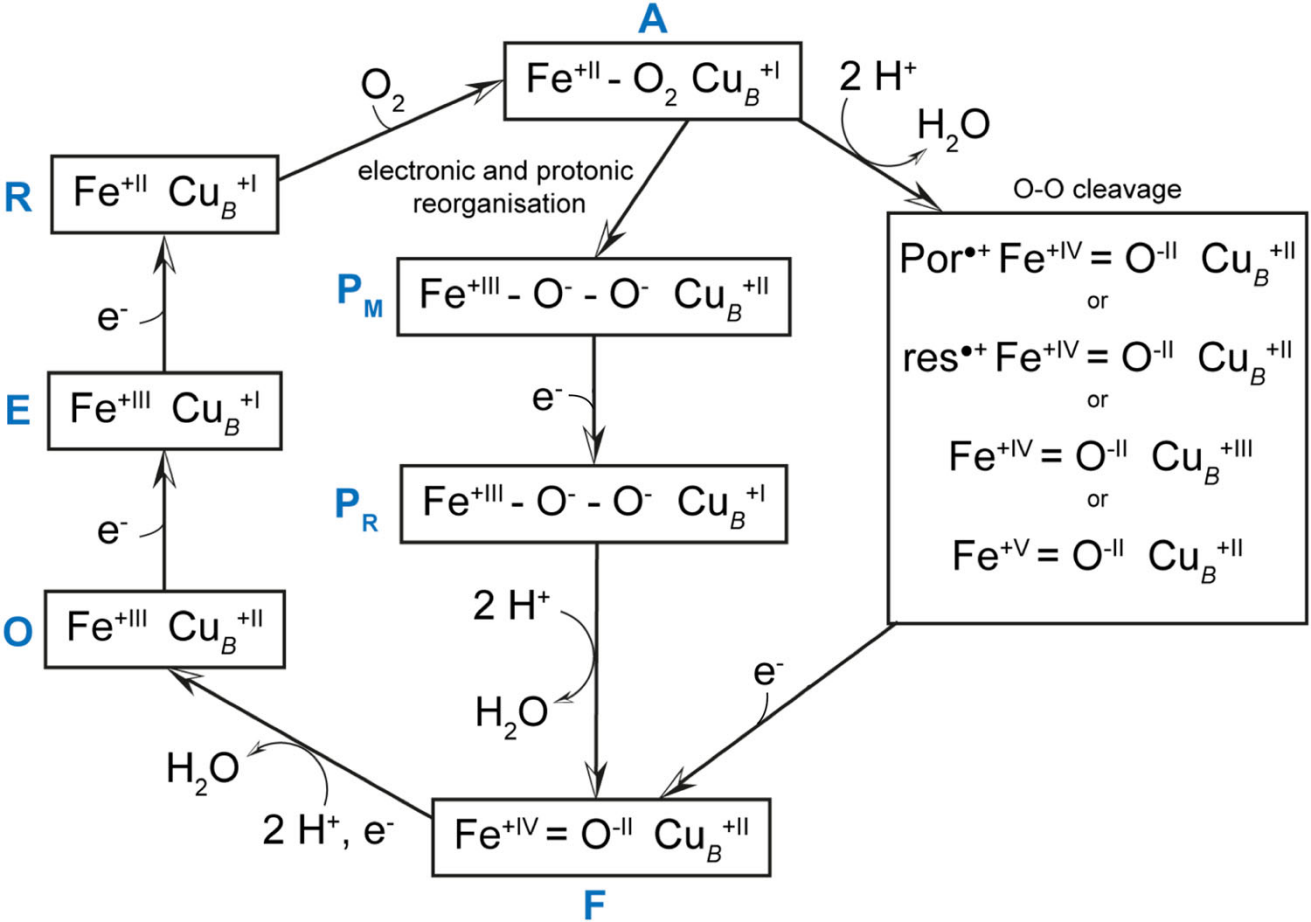
The structure of oxygen intermediates in the catalytic cycle of cytochrome *c* oxidase. As the chemical composition of the **P**-state remains unsettled, alternative proposed structures are presented (intact dioxygen bond left, split bond right). Por^**•**^ and res^**•**^ denote radicals of the porphyrin ring and a spatially close amino acid residue, respectively.

Cytochrome *c* oxidases (C*c*Os), members of the heme-copper superfamily of terminal oxidases, are among the most fundamental enzymes of life. Located in the inner membrane of mitochondria or of some prokaryotes they reduce molecular oxygen (dioxygen) to water and generate an electrochemical proton gradient across the membrane by using electrons from the external and protons from the internal membrane side as well as by proton pumping^1–3^. In order to understand these processes, it is essential to know the structures of the intermediates of the catalytic cycle which comprises the oxidized form (**O**-state), the reduced C*c*O (**R**-state), the **P**-state (after reaction with dioxygen), then the **F**-state and again the **O**-state after successive electron transfers (Fig. 1).

In the conventional view (see Fig. 1) the reaction catalyzed by C*c*Os starts with electron transfer from a reduced cytochrome *c* to Cu_A_. Electrons are subsequently transferred to the binuclear centre (BNC) via the low spin heme *a*. The BNC is composed of the high-spin heme *a*_3_ and the Cu_B_ center and constitutes the active site of the enzyme^4,5^. The oxidized BNC [**O** (Fe_*a*3_^3+^, Cu_B_^2+^)] is reduced by the first electron leading to the appearance of the **E**-state (***E*** = electronated) which is converted to an **R**-state [**R** (Fe_*a*3_^2+^, Cu_B_^+^)] by further electron transfer. The **R**-state binds dioxygen forming compound **A**^6^. The original believe that two electrons are transferred onto the bound dioxygen creating a peroxide in the BNC, the **P**-state, (see e.g., ref. ^7^) has been challenged on the basis of the results of resonance Raman spectroscopy and magnetic circular dichroism spectroscopy^8,9^. These results indicated that the dioxygen bond is already broken in the **P**-state, one oxygen atom has been bound to the heme *a*_3_-Fe via a double bond, thus forming an oxoferryl moiety whereas the second oxygen atom is released as water. However, for the splitting of the dioxygen bond four electrons are required. Two of them would be provided by the heme *a*_3_-Fe, one by Cu_B_. The potential donors of the missing fourth electron are shown (Fig. 1). Currently, a tyrosine residue cross-linked to a histidine ligand of Cu_B_ is favored. A simultaneous transfer of four electrons onto dioxygen is supposed to lead to the splitting of the dioxygen bond without the danger of forming reactive oxygen species. Whether a truly peroxidic **P**-state is part of the reaction cycle has been discussed controversially. Early Raman spectroscopy studies had suggested the presence of a peroxidic **P**-state intermediate while the later results have been interpreted in favor of the presence of an oxoferryl moiety in the BNC^8–10^. The proposal that the **P**-state already contains an oxoferryl moiety has found rapid acceptance because Weng and Baker had claimed that the 606- (**P**-state) and 580 nm (**F**-state) species do not differ in oxidation state, and the **F**-state, obtained after the input of the third electron, is generally believed to contain an oxoferryl moiety^11^. More recently there has been a comeback of a true peroxidic **P**-state intermediate^12^. Input of the fourth electron leads to formation of a water molecule (or a hydroxide) closing the cycle.

In this work we used single-particle cryo-electron microscopy (cryo-EM) to determine high-resolution structures of biochemically defined and adjusted intermediate states (**O, R, P**_**CO**_ (carbon monoxide induced)**, F**) of the wild type four-subunit C*c*O from *Paracoccus denitrificans* in lipid nanodiscs complexed with an Fv-antibody-fragment. These intermediate states refined locally to 1.9 Å (**O**), 2.6 Å (**R**), 1.9 Å (**P**_**CO**_) and 2.3 Å (**F**) resolution. Our density maps provided insights into the active site configuration during the catalytic cycle revealing expected and unexpected rearrangements of dioxygen species in proximity to the BNC for the **O**, **P,** and **F** intermediates. In addition, we found densities for many dioxygen molecules in particular in the **O**-state C*c*O. Collectively, our results suggest that the established C*c*O catalytic cycle may have to be revised.

## Results and discussion

**Fig. 2:**
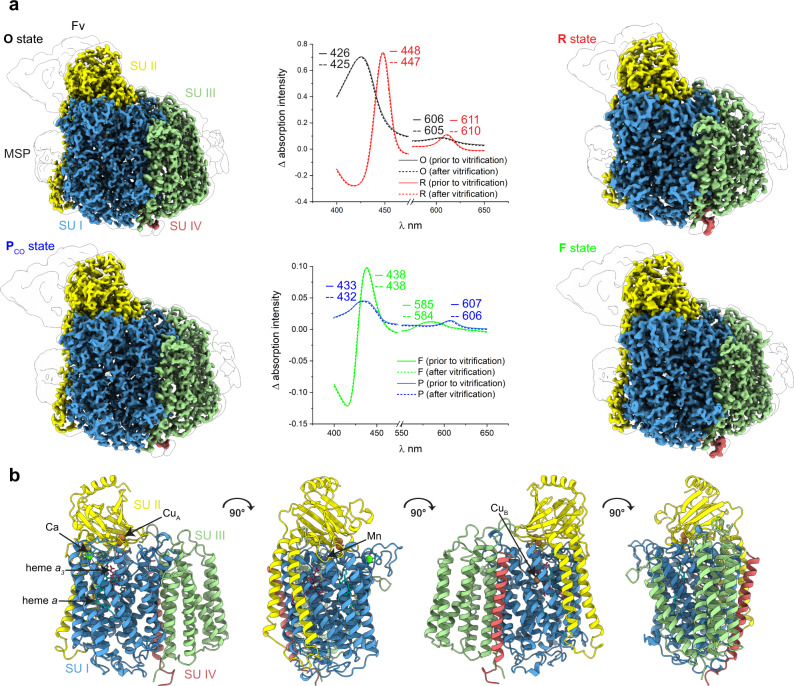
Cryo-EM structure of the *aa*_*3*_ C*c*O oxidase from *P. denitrificans*. **a** Surface representation of each intermediate state of the CcO at 1.9 Å (**O**), 2.6 Å (**R**), 1.9 Å (**P**_**CO**_), and 2.3 Å (**F**) resolution. Uv/visible absorption spectra were individually and consistently recorded (intermediate state minus **O**-state). The illustrated map densities are filtered to equal contour levels of 1. **b** Ribbon representation of all four subunits with corresponding cofactors of the C*c*O. Color scheme: SU I, blue; SU II, yellow; SU III, green; SU IV, red. The antibody fragment (Fv) and membrane scaffold protein (MSP) are shown as a white surface.

We recorded UV/visible absorption spectra throughout the specimen preparation to confirm the presence of the desired specific electron-induced catalytic intermediate states (Fig. 2, Supplementary Fig. 1). The **O**-state C*c*O showed a Soret peak with an absorption maximum at 426 nm, while that of the **R**-state is red-shifted to 448 nm. The difference absorption spectrum (**P**_**CO**_ minus **O**) for the two-electron induced **P**_**CO**_-state (CO + 2OH^–^ → 2e^–^ + CO_2_ + H_2_O) showed a Soret peak at 433 nm (α-band at 607 nm) which is distinct from the three electron-induced F state with its maximum at 438 nm (α-band at 585 nm).

### Identification of the dioxygen channel

**Fig. 3:**
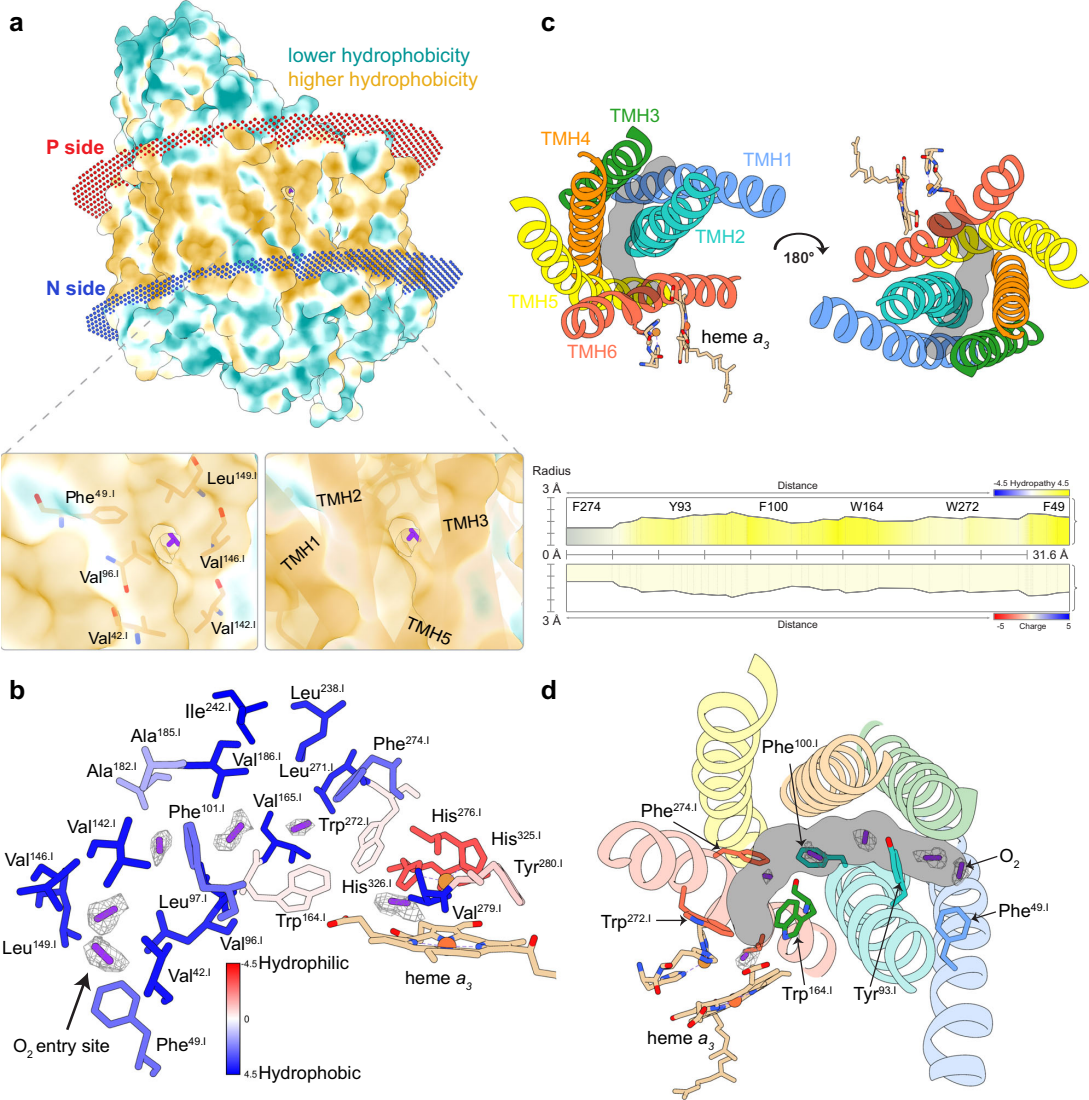
Oxygen entry site and channel composition. **a** Surface representation of C*c*O and its oxygen entry point located between the transmembrane helix (TMH) 1 and 3 in the hydrophobic transmembrane site. The illustrated map density is filtered to a contour level of 2. **b** Detailed composition of the oxygen-conducting channel based on their hydrophobicity. The illustrated map density is filtered to a contour level of 0.5. **c** Interior channel embedded in between six transmembrane helices and channel lining residues predicted by Mole 2.5. **d** Overlay of channel prediction and individually displayed lining residues with corresponding dioxygen densities. Dioxygen molecules are shown as purple sticks, the corresponding densities are highlighted by mesh volumes.

We start with a description of the **O-**state (Supplementary Fig. 2–7). Our density map allowed us to model a continuous cluster of dioxygen molecules, starting at the hydrophobic membrane interface near Val^42.I^ of TMH1.I and Leu^149.I^ of TMH3.I, ending at Val^279.I^ in proximity to the dioxygen binding site of heme *a*_3_ and Cu_B_ (Fig. 3a/3b). Comparable corresponding densities were not observed for the fully reduced enzyme, however, they are in excellent agreement with the molecular dynamics results of Hofacker and Schulten^13^. We performed computational assisted calculations (MOLE 2.5^14^) to map possible channels within the **O**-state structure. We identified a continuous hydrophobic pathway that overlaps with the densities assigned as oxygen molecules (Fig. 3c/3d). This pathway is in good agreement with the previously characterized oxygen channel of a B-type cytochrome *c* oxidase^15^. The entry site of our proposed channel is defined by a cavity at the interface between the transmembrane region and the hydrophobic membrane environment located in subunit I. Site-directed mutagenesis experiments have demonstrated a significantly higher K_*M*_ for oxygen when replacing Val^279.I^ by isoleucine^16,17^. We conclude that impairment of oxygen binding and reduction in the V279I mutant is caused by the introduced bulky residue at the end of our identified oxygen channel restricting access to the dioxygen reduction site (Supplementary Fig. 8).

### O-state

**Fig. 4:**
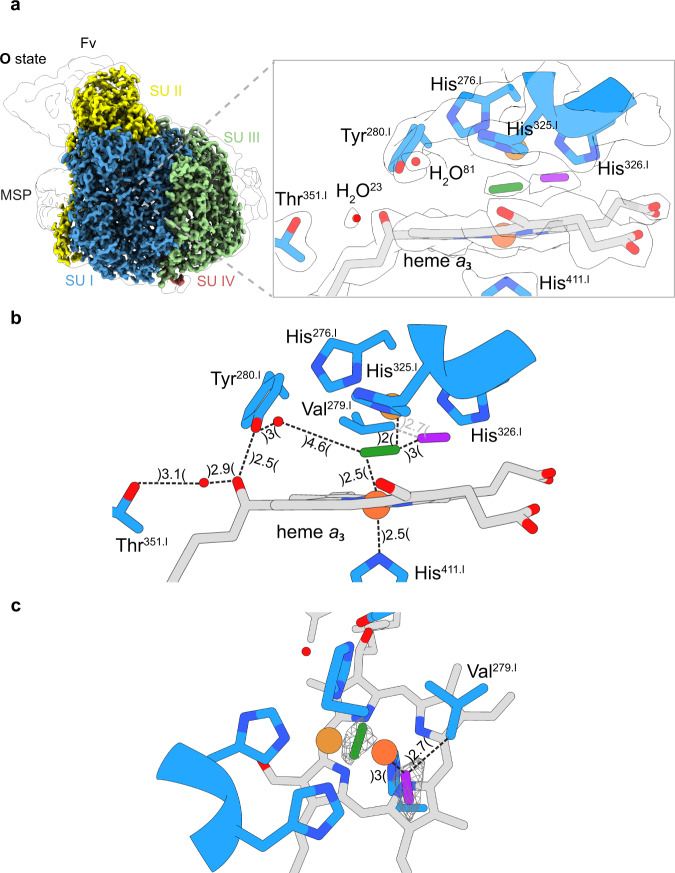
Cryo-EM density map (O-state) and modelled structure of the binuclear site in C*c*O. **a** Structural overview of the binuclear site in the **O**-state. The bridging peroxide species (green) is located in-between the iron (orange) and the copper (brown) atom. In close proximity to the observed bridging ligand, a second prominent density presumably molecular oxygen (purple), is located. **b** Measured distances (Å) for the modelled molecules at the active site. **c** Top view of the active site. The corresponding density map is illustrated as a white surface or mesh volumes. The illustrated map densities are filtered to equal contour levels of 1.5.

In the **O**-state structure, we were able to identify a prominent density within the binuclear center (BNC) between heme *a*_3_ and Cu_B_. Careful refinements with respect to distances to neighbouring molecules led to the assignment of a bridging peroxide species for this density in agreement with previous X-ray structures (Fig. 4)^18–22^. As previously outlined, more than 90 non-protein-derived peroxide structures that bridge two metal ions include an average length of 1.44 ± 0.06 Å for the average O-O the distance which is in accordance with our fitted peroxide dianion (1.42 Å)^20^. Neither a single nor two hydroxide ions could efficiently reside in this location since the density of one anion would be too weak while individual hydroxide ions couldn’t be accommodated due to insufficient space in proximity to both metals. The Fe_*a3*_-Cu_B_ distance is 4.7 Å with the heme *a*_*3*_ iron (93°) coordinated almost in-plane to the porphyrin scaffold calculated from the merged bond angles heme *a*_*3*_ N-Fe-His_411_. The existence of peroxide in the BNC as observed in various X-ray structures has not found much acceptance despite the fact that it has been shown that six electrons are required to fully reduce the as isolated oxidized C*c*O^18,21,23^. Four electrons would be needed to reduce the four prosthetic groups of C*c*O and two to reduce the peroxide. A peroxide bridge would be ideally suited to electrostatically compensate the positive charges at Fe_*a3*_ and Cu_B_. The peroxide has been claimed to be an artifact of X-ray radiation by X-ray induced reduction of dioxygen molecules present in the crystals^21,23,24^. The observation of a density for a dioxide molecule, presumably peroxide, by cryo-EM definitely rules out such explanations. In close proximity to the peroxide dianion (3 Å), we observed a distinct density feature which most likely represents a dioxygen molecule bound near Val^279.I^ (2.7 Å). This resting oxygen molecule is located at the end of the identified substrate conducting channel presented above.

### R-state

**Fig. 5:**
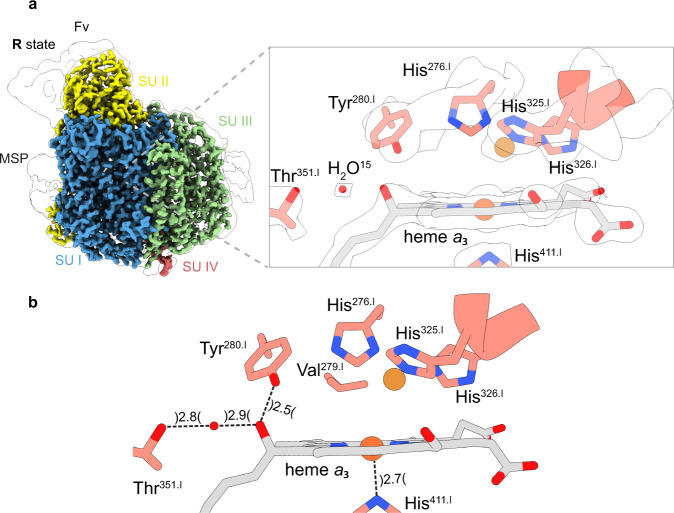
Cryo-EM density map (R-state) and modelled structure of the binuclear site in C*c*O. **a** Structural overview of the binuclear site in the **R**-state. Densities located between the iron (orange) and copper atom (brown) were not observed, indicating a fully reduced binuclear site. **b** Measured distances (Å) for the modelled molecules at the active site. The corresponding density map is illustrated as a white surface. The illustrated map densities are filtered to equal contour levels of 1.5.

**Fig. 6:**
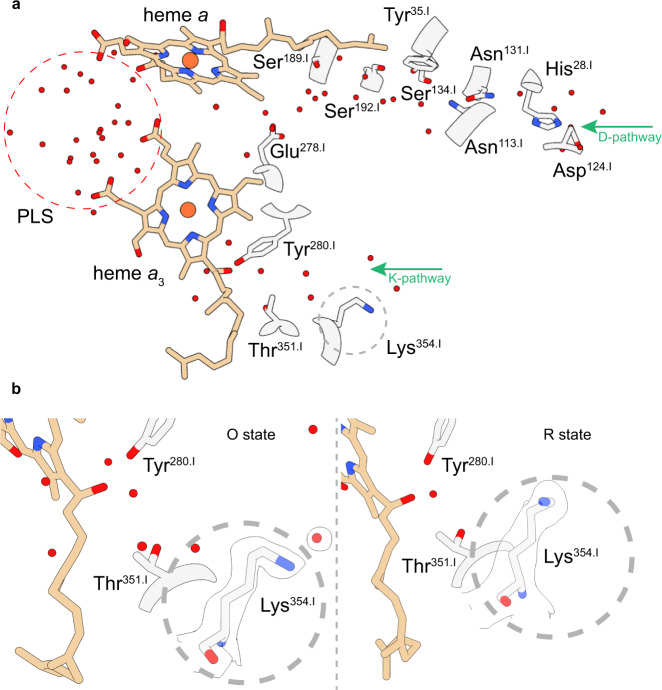
Proton transfer pathways in C*c*O. **a** A structural overview of the two proton transfer pathways. The K and D-pathway are defined by their respective amino acids. Several water molecules could be assigned in each pathway and an accumulated water cluster could be seen near the A-ring propionate of heme *a*_3_, presumably the proton-loading site (PLS). **b** A redox-dependent positional change of the side chain of the key residue Lys^354.I^, located in the K-pathway, was observed. It looks as the reduction of the BNC leads to an electrostatic attraction of the potentially positively charged side chain of Lys^354.I^. The corresponding density map is illustrated as a white surface. Illustrated map densities are filtered to equal contour levels of 1.

The structure of the fully reduced **R**-state C*c*O does not show any density between Fe_*a*3_ and Cu_B_ nor does it show densities for bound oxygen in the substrate conducting channel (Fig. 5). The absence of the densities assigned to dioxygen molecules in the **O**-state after the dithionite treatment supports the assignment of the observed densities in the channel of the **O**-state to dioxygen molecules. An interesting distinction between the **O**-state and the **R**-state is the different side-chain orientation of the prominent residue Lys^354.I^ of the K-pathway (Fig. 6). In the fully reduced **R**-state the side chain is found closer to the then more negatively charged BNC that might indicate that the lysine side chain is protonated, or becomes protonated during reduction. Figure 6 also presents the water molecules which form part of the proton transfer network and of the pump loading site.

### P-state

**Fig. 7:**
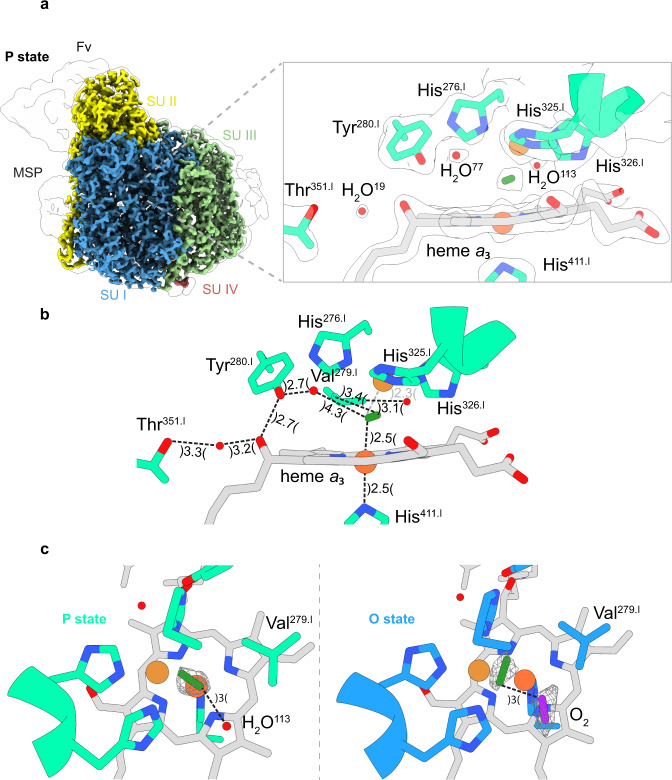
Cryo-EM density map (P-state) and modelled structure of the binuclear site in C*c*O. **a** Structural overview of the binuclear site in the **P**-state. The bridging dioxide ligand (green) is embedded between the iron (orange) and the copper (brown) atom. The previously observed dioxygen molecule in the **O**-state near Val^279.I^ is replaced by a water (H_2_O^113^). **b** Measured distances (Å) for the modelled molecules at the active site. **c** Top view of the binuclear center of the **O** and **P**-state to illustrate the rotation of the bridging dioxide species. The corresponding density map is illustrated as a white surface or mesh volumes. Illustrated map densities are filtered to equal contour levels of 1.5.

**P**-states can be induced by a number of different methods^25^. We chose to generate the **P**-state by limited exposure to carbon monoxide in the presence of oxygen thus denoted here as **P**_**CO**_-state. This is a classical procedure discovered by Peter Nicholls and was used also in previous experiments^25,26^. The presence of the **P**_**CO**_-state was unambiguously confirmed by uv/vis spectroscopy. The structure in and around the BNC, obtained at a local resolution of sub 2 Å, met us with a big surprise. We identified a specific density between the two metal centers of the active site while, compared to the **O**-state, the nearby dioxygen molecule appears to be absent and to be replaced by a water molecule or a hydroxide ion (Fig. 7). The density map indicates the presence of a diatomic species, most likely with an intact O-O bond in the **P**_**CO**_-state. Based on the location of this density and due to geometrical and steric restraints, we can exclude the presence of an oxoferryl heme species, and of a hydroxide ion or water bound to Cu_B_ (Cu_B_ – OH^−^/H_2_O) suggested by previous studies. The two oxygen atoms would come too close. The interatomic Fe-Cu_B_ distance is 4.7 Å while the heme *a*_3_ iron remains almost in the plane with the ring system. The overall similarity to the **O**-state is extreme with the exception of the crosslink distance N_ε2-_His^276.I^ - C_ε2-_Tyr^280.I^, decreasing from 2.3 Å (**O**) to 1.6 Å (**P**). Movements of the related helices are not observed (Supplementary Fig. 9). The density for the diatomic species in the **P**-state appears to be more parallel to the heme *a*_3_ macrocycle than in the **O**-state. As outlined above the view based on resonance Raman spectroscopy that the dioxygen double bond has been already broken in the **P**-state appears to be widely accepted. However, one has to note that the species providing the signal in Raman spectroscopy may not reflect the most abundant population in the sample^27^. In contrast, particle averaging and class sorting in cryo-EM result in weighted map densities with the most populated species having the strongest contribution to the map features.

What about the chemical experiment providing evidence for a split dioxygen bond in **P**_**CO**_-state? There, the **P**_**CO**_-state was generated by flashing off carbon monoxide from mixed-valence CO bound C*c*O in the presence of ^18^O_2_. Approximately half of the ^18^O-label, compared to the amount of **P**_**CO**_-state C*c*O, was found as water^28^. However, the reaction of CO with dioxygen yielding CO_2_ and water would provide the same experimental result.

What then can be the nature of the diatomic species between the two metals of the BNC? We can exclude carbon monoxide on the basis of the spectroscopic features of the state generated and investigated. A peroxide dianion would be a possibility. However, the BNC of the **O**-state contains two electrons more than that of the **P**_**CO**_-state (see Fig. 1). If one accepts that the **O**-state BNC contains a peroxide dianion, the BNC of **P**_**CO**_-state should contain a neutral dioxygen molecule! In agreement with this proposal, quantum chemical calculations have indicated that binding of dioxygen to the oxidized BNC is energetically favorable^23^.

### F-state

**Fig. 8:**
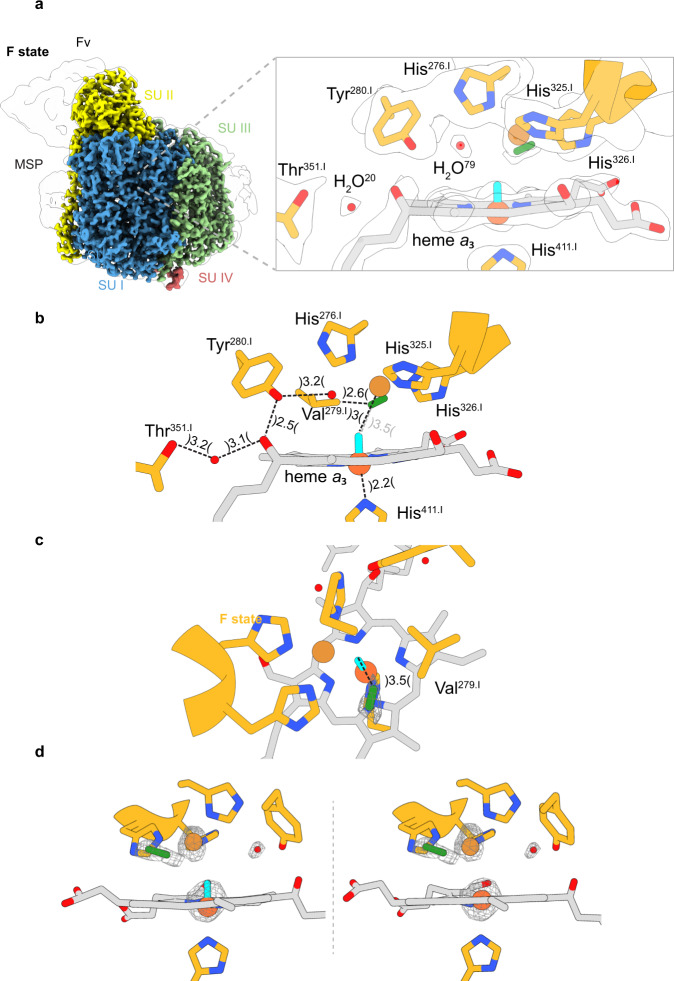
Cryo-EM density map (F-state) and modelled structure of the binuclear site in C*c*O. **a** Structural overview of the binuclear site in the **F**-state. No bridging ligand is located between the iron (orange) and the copper (brown) atom. Instead, the density map may indicate the presence of an oxoferryl (cyan) at the heme and a dioxide species (green) in close proximity. **b** Measured distances (Å) for the modelled molecules at the active site. **c** Top view of the binuclear center of the **F**-state. **d** Modeling of the iron of heme *a*_3_ with a bound oxygen (oxoferryl) and without. The corresponding density map is illustrated as a white surface or mesh volumes. Illustrated map densities are filtered to equal contour levels of 1.5.

In order to gain insights into the previously unknown ligand arrangement of the **F-**state BNC, we treated the C*c*O with an excess of hydrogen peroxide as published^25^. We obtained the structure of the **F-**intermediate at a resolution of 2.3 Å. The space between Cu_B_ and heme *a*_3_ does not contain a distinct density (see Fig. 8) as observed for the **P**_**CO**_ state (Fig. 7). Hence, the absence of a dioxygen species is apparent. Based on the expectations we modelled a geometrically optimized oxoferryl group in the center of heme *a*_*3*_ in agreement with previously postulated structures, despite the fact, that established X-ray crystallographically determined electron densities of oxoferryl heme moieties in proteins reveal a much better shape for the oxoferryl moiety^29^. However, modeling simply a ferric iron into the central heme *a*_3_ density is at least equally well possible (Fig. 8d). Modelling of a water molecule as a potential fourth Cu_B_ ligand, providing a hydrogen bond to the oxoferryl oxygen, was not possible because of insufficient space. The Fe_*a3*_-Cu_B_ distance of 4.5 Å is similar to that in the **O**/**P**_**CO**_ state while the coordinated iron is slightly more displaced from the center of the heme macrocycle (95°) compared to the **O** state (93°). Furthermore, the cross-linked His^276.I^ - Tyr^280.I^ distance has increased by more than 1 Å (2.7 Å) compared to the **P**_**co**_ state (1.6 Å). Clearly, the covalent crosslink between the N_ε2_ of His^276.I^ and C_ε2_ of Tyr^280.I^ appears to be subject of structural rearrangements based on the interatomic distance. This observation also means that a reinvestigation of whether the crosslink is opened in the **F**-state is required. In addition, the bonding distance between His^411.I^ – Fe_*a3*_ is shortened by ~0.3 Å (2.2 Å) which can be explained by a different protonated state as outlined previously^30^. Intriguingly, a prominent and undescribed density is located between His^326.I^ and Val^279.I^ in close proximity to Cu_B_ (3.3 Å). Its shape and location argue for a dioxide species, dioxygen or superoxide. After reduction the latter could form a peroxide bridge between Fe_*a3*_ and Cu_B_ as observed in the **O**-state. We find a similar but slightly more displaced density also in the **O**-state in proximity to Val^279.I^ in a more distant position (~0.4 Å, 3.7 Å) towards the Cu_B_. The presence of superoxide at this position in the **F**-state agrees well with complementary EPR and ligand-mimicking studies, which have shown that treatment of the **F-**state C*c*O with catalase leads to an **O**-state like UV/vis spectrum and the appearance of a tyrosine radical^25^. This observation can be explained if a superoxide present takes an electron from the C*c*O thus being converted to a peroxide that leaves the enzyme and is split by the catalase.

As mentioned above and presented in Fig. 8, modelling of the central density of heme *a*_3_ iron as a ferric iron atom is at least equally well possible. The experimental result mentioned above that treatment of the **F**-state C*c*O with catalase leads to the formation of a C*c*O form with UV/vis spectral properties very similar to the **O**-state^25^. The only difference to the **O**-state spectrum is the appearance of a broad absorption band around 630 nm. This band has been considered to be a charge transfer band of the ferric heme *a*_3_ iron^31^. How could the simple addition of catalase lead to the removal of an oxygen atom from a central ferryl iron? We also would like to remind the reader of the postulate by Weng and Baker that the 606- (**P**-state) and 580 nm (**F**-state) species do not differ in oxidation state^11^.

The scenario which fits our experimental results best is the following: The classical **P**-state is an oxygenated oxidized form of C*c*O. Upon reduction of the bound dioxygen by one electron a superoxide anion is formed which is observed near Cu_B_. This form corresponds to the classical **F**-state. Upon input of another electron, the superoxide is reduced to a peroxide, leading to the **O**-state with the peroxide bridging the heme *a*_3_ iron and Cu_B_.

**Fig. 9:**
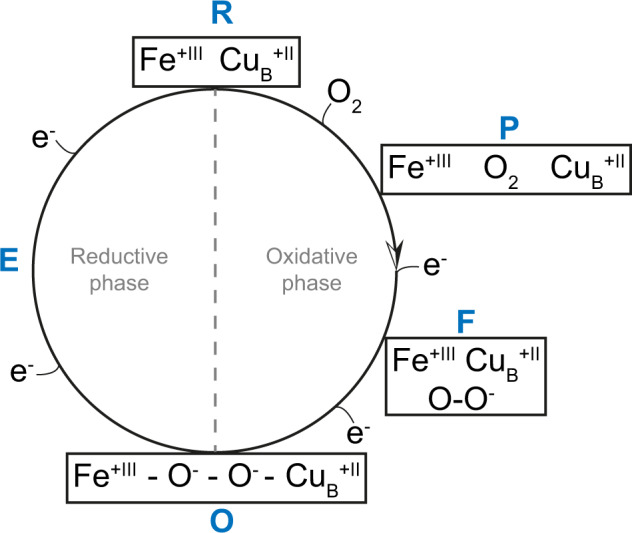
A potential catalytic cycle of C*c*O based on our results. The classical **P**-state is an oxygenated oxidized form of C*c*O. Input of one electron reduces the bound dioxygen to superoxide which remains bound in the BNC near Cu_B_ (classical **F**-state). Input of another electron reduces the superoxide to a peroxide which forms a bridging ligand in the BNC of the **O**-state. The next input of an electron leads to the formation of the classical **E**-state whose structure is still unknown. Input of the fourth electron leads to the formation of a 2-electron **R**-state which binds a dioxygen molecule closing the cycle. Two water molecules or one water molecule plus one hydroxide ion are formed during the input of the third and fourth electrons in the classical reductive phase.

For a complete cycle (Fig. 9) the observed **O**-state has to be reduced by two more electrons which would lead to the formation of two water molecules (or one water molecule and one hydroxide ion) which would have to be replaced by a dioxygen molecule forming the **P**-state again. We look with great interest into the structure determination of the one-electron reduced **E**-state.

## Methods

### Production of cytochrome *c* oxidase from *Paracoccus denitrificans*

The cytochrome *c* oxidase (cytochrome *aa*_3_) from *Paracoccus denitrificans* was produced in P. denitrificans AO1 cells transformed with the pUB39 (pBBR1 MCS derivative) plasmid and RP-4 helper strain via triparental mating according to a standardized in-house protocol^32,33^. A pre-culture was set up by adding 0.1 ml of 50% glycerol stock to 50 ml succinate (25 µg/ml kanamycin, 25 µg/ml streptomycin) growth medium. Cells were incubated at 32 °C while shaking (180 rpm) overnight. An intermediate-culture was started by inoculating 500 ml succinate (25 µg/ml kanamycin, 25 µg/ml streptomycin) growth medium with 50 ml of the pre-culture. Subsequently, cell production was carried out by growing the main culture with 2.5 l by adding 200 ml from the intermediate-culture at 32 °C while shaking (150 rpm) for approximately 8 hours. After harvest and homogenization, cells were disrupted using a high-pressure homogenizer (Constant Systems Ltd.) with an applied pressure of 25 kpsi. The cell lysate was centrifuged at 6,000 × g at 4 °C for 60 m followed by an ultra-centrifugation step with a speed of 205,000 × g at 4 °C for 12 h. Collected membrane pellets were suspended in 50 mM KPi (pH 8) with a concentration of 20 mg/ml.

### Production and purification of the antibody-fragment from *E. coli* JM83 pASK68

The antibody-fragment Fv7E2 was produced in E. coli JM83 with the plasmid pASK68 which possess the ability to bind to SU II of CcO and carries a C-terminal Strep-tag^34,35^. For production, a pre-culture was inoculated with a volume of 0.1 ml 50% glycerol stock and grown in a 200 ml LB (100 µg/ml ampicillin) growth medium at 30 °C (180 rpm) overnight. The 2 L main culture (100 µg/ml ampicillin) was grown at 23.5 °C while shaking (165 rpm) to an OD600 of 0.5 before recombinant antibody-fragment production was induced by the addition of 0.5 mM IPTG for additional 3 h. Collected cell material was centrifuged at 3500 × g for 15 m at 4 °C and pellets were subsequently mixed with 500 mM sucrose, 35 mM KPi (pH 8) for 20 m on ice. The disrupted cell lysate was centrifuged at 24,000 × g at 4 °C for 60 m and the supernatant was collected.

### Purification of cytochrome *c* oxidase from *P. denitrificans*

For preparation of the four-subunit cytochrome *c* oxidase, pelleted membranes were solubilized at pH 8 with 1 % dodecyl-β-maltoside (DDM) with a resulting mass ratio of 1 mg detergent per 5 mg of membrane proteins. In a first step, solubilized C*c*O was incubated with the strep-tagged Fv fragment for 30 min at 4 °C while stirring to allow efficient binding. Final mixture was centrifuged at 235,000 × g for 60 m at 4 °C and the supernatant was loaded on a Strep-Tactin^®^XT Superflow^®^ high capacity column (IBA GmbH) to perform affinity chromatography. For washing during purification, 5 column volumes [CV] (50 mM KPi pH 8, 0.05% DDM) were used and for the final elution (5 CV), the washing buffer was supplemented with 50 mM biotin. To increase sample homogeneity, ion-exchange chromatography was performed using a Q Sepharose^®^ High-Performance column (Sigma Aldrich). Two steps of washing containing 50 mM/200 mM KPi (pH 8), 0.05% DDM were performed before eluting with 300 mM KPi (pH 8) and 0.05% DDM. As a final sample polishing step, size exclusion chromatography was done using a Superdex 200 10/300 increase column (GE Healthcare Life Sciences) with a running buffer consisting of 50 mM KPi (pH 8) and 0.02% DDM. Each purification was done in the presence of K_3_[Fe(CN)_6_] to keep the redox state of the cytochrome *c* oxidase consistent.

### Production and purification of MSP1D1

The production of the N-terminal 7x histidine-tagged MSP1D1 (Membrane scaffold protein) was done according to a standardized protocol^36^. Therefore, BL21Gold (DE3) *E. coli* cells were transformed with pMSP1D1 and grown in LB-Kan medium (50 µg/ml kanamycin) at 37 °C. By reaching an OD_600_ of 0.6, induction with 1 mM IPTG was carried out to start the MSP1D1 production for 4 h before cell harvest. The pellet from 4 L cell culture was resuspended in a cell disruption buffer (20 mM Tris-Cl pH 8, 1 mM PMSF) and afterwards supplemented with Triton X-100 (1% (v/v)). The resulting lysate was centrifuged and filtered. In a final step, the cell solution was supplemented with 500 mM NaCl. The lysate was mixed with 6 mL Ni-NTA agarose (Thermo Fisher) which had been equilibrated with the washing buffer (20 mM Tris-Cl pH 8, 500 mM NaCl, 1% Triton X-100). The mixture was incubated for 12 hours while stirring at 4 °C. Next, the solution was loaded, washed and eluted with 500 mM imidazole. Eluted fractions were pooled and dialyzed against buffer (20 mM Tris-Cl, pH 7.5, 150 mM NaCl) and finalized samples were concentrated to 5 mg/mL, frozen in liquid nitrogen and stored at –80 °C.

### Reconstitution of cytochrome *c* oxidase in MSP1D1 and lipids

The reconstitution of cytochrome *c* oxidase into lipid nanodiscs was accomplished by using the aforementioned protocols^36^. Here, mixing POPC, MSP1D1 and the C*c*O was done in a molar ratio of 500:10:1 and subsequently incubated at 4 °C for 1 h. Detergent removal from the mixture was conducted by adding three times 0.2 mg dried Bio-Beads™ SM-2 (Bio-Rad) every 2 h keeping at 4 °C. For successful formation, the mixture was incubated for an additional 12 h at 4 °C while shaking. Removal of Bio-Beads was done by filtration and the reconstituted C*c*O was applied to a Superdex 200 10/300 increase column to exclude empty nanodiscs using 50 mM KPi (pH 8) as a final buffer. Fractions containing nanodisc-reconstituted *aa*_*3*_ oxidase were pooled together, concentrated to 2 mg/ml and directly used for subsequent cryo-EM experiments.

### Optical spectroscopy

To establish a defined ground state with an Soret maximum around 425 nm, the protein was fully oxidized during the size exclusion chromatography step in the presence of 200 µM K_3_[Fe(CN)_6_]. Uv/visible absorption spectra of each state were recorded using a PerkinElmer Lambda 35 UV/visible spectrophotometer. Different biochemical defined states (**R, P, F**) were set according to a given in-house method with slight adjustments^25^. 50 mM KPi (pH 8) was mixed with 2 mg/ml (~12 µM) of reconstituted C*c*O in a quartz cuvette with 1 cm path length. To prepare the artificial intermediates **P**_**co**_ and **F**, the reconstituted C*c*O was either incubated with CO (Air Liquid Deutschland GmbH) aerobically for 15 m on ice while stirring **(P**_**CO**_**)** or mixed with H_2_O_2_ in 1:500 molar ratio **(F)**. Full reduction (**R**) of the protein mixture was achieved by adding stoichiometric amounts of sodium dithionite. To confirm the presence of each electron-induced state, UV/visible absorption spectra were recorded prior to sample vitrification and afterwards for each cryo-EM experiment from the same protein batch. From sample adjustment to freezing in liquid ethane, no more than 10 m were used to avoid potential decay processes.

### Single-particle cryo-electron microscopy

#### Sample vitrification

Quantifoil R1.2/1.3 copper grids (mesh 200) were firstly washed with chloroform and then glow discharged twice with a PELCO easiGlow device at 15 mA for 45 s. For each recorded data set, a volume of 4 µl protein sample (2 mg/ml) was applied on the grid before plunge freezing. Samples were vitrified at 4 °C, 100% humidity, and a blot force of 20 using a Vitrobot IV device (Thermo Fisher Scientific). The blotting time was set to 4 s before plunge freezing in liquid ethane.

#### Image recording

Images for each data set were recorded by using a Titan Krios G3 microscope operated at 300 kV (Thermo Fisher Scientific). Coma and beam tilt correction was done by using EPU (Thermo Fisher Scientific) and data collection was conducted by operating in an electron counting mode with a Falcon III direct electron detector at a nominal magnification of x96,000, corresponding to a calibrated pixel size of 0.833 Å. An accumulated dose of 0.98 e^–^/Å^2^ per fraction was used for the 30 dose-fractionated frames (~ total dose 30 e^–^/Å^2^). Defocus values were applied in a range from 0.5 to 2.5 µm.

#### Image processing

Collected data in MRC format were consistently processed with RELION-3.1^37,38^ and motion-corrected with the MotionCor2 algorithm^39^. Initial CTF parameters from each dose-weighted image were determined by using CTFFIND4^40^ and particle images were automatically selected followed by initial model building, 3D classification, CTF refinement, Bayesian polishing and final map reconstruction using RELION-3.1. Unfiltered maps were sharpened by applying different b-factors for building and visual improvement purposes. The final overall resolution was estimated by using the gold-standard Fourier shell correlation (FSC_0.143_) which was calculated from two independently refined data sets. Global resolution anisotropy was examined by RELION-3.1. Observable map quality improvements were achieved by applying a density-modification procedure to each data set in a consistent way^41^. For this purpose, both half maps and a sequence file were used together with a model file, a full map file and a mask file. Significant map improvements were already observed after one cycle and were repeated until no further improvement was observed (Supplementary Figs. 2–7).

### Model building and geometry refinement

For building the four-subunit atomic model of the cytochrome *aa*_*3*_ oxidase, the Protein Data Bank (PDB) submission by Harrenga et al. (PDB 1QLE) was used as a template structure^42^. After manual backbone fitting and correct fitting of side chains in the respective map densities, real-space refinement was done using Phenix (version 1.18)^43^. As a final building step, density-modified maps were used for more precise molecule allocation around the catalytic center. Each finalized model was validated by the MolProbity online server^44^. A final summary for each model parameter and its corresponding cryo-EM statistics can be found in Supplementary Table 1. Finalized models were visualized by using Chimera^45^ and ChimeraX^46^.

#### Visualization of an oxygen channel

The proposed oxygen channel was mapped with MOLE 2.5 (bottleneck radius: 1.5 Å, bottleneck tolerance 3 Å, origin radius 5 Å, surface cover radius 10 Å, max tunnel similarity 1)^14^ and visualized with Chimera. Oxygen molecules were manually placed with Coot into corresponding densities observed in the O-state map.

### Reporting Summary

Further information on research design is available in the Nature Research Reporting Summary linked to this article.

## Supplementary information

Supplementary Information

Reporting Summary

## Data Availability

The generated cryo-EM maps of the *aa*_*3*_ cytochrome *c* oxidase from *Paracoccus denitrificans* have been deposited at the Electron Microscopy Data Bank under accession codes EMD-11921, EMD-11922, EMD-11924 and EMD-11925. The models of the *aa*_*3*_ cytochrome *c* oxidase structures were submitted to the PDB data bank with the accession numbers: 7ATE, 7AU6, 7ATN, and 7AU3. 1QLE was used as a template. All other data is presented in the main text or supplementary information.
