# Supplementary material for: Cryo-EM structures of intermediates suggest an alternative catalytic reaction cycle for cytochrome c oxidase
Source: Nat Commun. 2021 Nov 25;12:6903. doi: 10.1038/s41467-021-27174-y (PMC8617209; doi:10.1038/s41467-021-27174-y)
Supplement: Supplementary file 1 — Supplementary Information [file 41467_2021_27174_MOESM1_ESM.pdf]

Supplementary Information

Cryo-EM structures of intermediates suggest an alternative catalytic reaction cycle for cytochrome *c* oxidase

Felix Kolbe[1†], Schara Safarian[1†], Żaneta Piórek[2], Sonja Welsch[3,] Hannelore Müller[1], Hartmut Michel[1*]

Correspondence to: (hartmut.michel@biophys.mpg.de)

This PDF file includes:

Supplementary Table 1

Supplementary Fig. 1-9

Supplementary Table 1. Cryo-EM data collection and validation. Cryo-EM data statistics of the **O**, **R**, **P** and **F**-state of the cytochrome *c* oxidase from *P. denitrificans*

	**O-state**	**R-state**	**P-state**	**F-state**
**Data collection**				
Accession number	EMD-11925	EMD-11922	EMD-11921	EMD-11924
Magnification	96k	96k	96k	96k
Voltage / kV	300	300	300	300
Dose / e^{-} Å^{-2}	30	30	30	30
Pixel size / Å	0.833	0.833	0.833	0.833
Defocus range / µm	-0.5 to -2.5	-0.5 to -2.5	-0.5 to -2.5	-0.5 to -2.5
Recorded movies	5140	5835	4487	4613
Final particle images	234,751	289,627	268,222	207,736
Camera	Falcon III	Falcon III	Falcon III	Falcon III
Microscope	Titan Krios G3	Titan Krios G3	Titan Krios G3	Titan Krios G3
**Image processing**				
Initial model	*de novo* generated with RELION 3.1, filtered to 15 Å			
Resolution ($FSC_{0.143}$) / Å	2.4	2.66	2.37	2.57
Applied B-factor / Å^{2}	-100	-120	-94	-109
Density modified resolution / Å (FSC_{ref})	1.9	2.5	1.9	2.3
**Model refinement**				
PDB accession	7AU6	7ATN	7ATE	7AU3
Validation				
$FSC^{map\text{-}to\text{-}model}_{(0.5)}$ / Å	1.9	2.6	1.9	2.3
MolProbity score	2.25	2.10	2.1	1.88
Composition				
Atoms	9024	8944	9056	9012
Protein residues	1092	1098	1098	1096
Waters	148	83	145	162
Ligands	2 PC1, 2 HEA, 1 CUA, 1 MN, 1 CA, 1 CU, 1 PEO, 6 OXY	1 PC1, 2 HEA, 1 CUA, 1 MN, 1 CA, 1 CU	1 PC1, 2 HEA, 1 CUA, 1 MN, 1 CA, 1 CU, 1 PGV, 1 PEO	2 HEA, 1 CUA, 1 MN, 1 CA, 1 CU, 1 PGV, 1 OX, 1 2FK
Bonds (R.M.S.D.)				
Length (Å)	0.013	0.012	0.010	0.016
Angles (°)	1.576	1.136	1.135	1.961
B-factors (min/max/mean)				
Protein	5.98/15.06/9.19	4.30/35.23/14.04	7.1/27.89/14.06	3.48/10.9/5.58
Ligand	6.12/15.82/10.23	5.01/41.57/19.82	8.96/24.67/15.79	4.58/15.42/6.97
Waters	6.75/10.13/8.52	5.64/15.48/9.49	8.51/16.13/11.91	3.7/6.35/5.18
Clashscore	18.54	14.32	16.89	13.09
Ramachandran plot (%)				
Favored	96.49	96.51	96.79	97.70
Allowed	3.51	3.39	3.21	2.11
Outliers	0	0.09	0	0.18
Rotamer outliers (%)	1.9	1.89	1.67	1.78

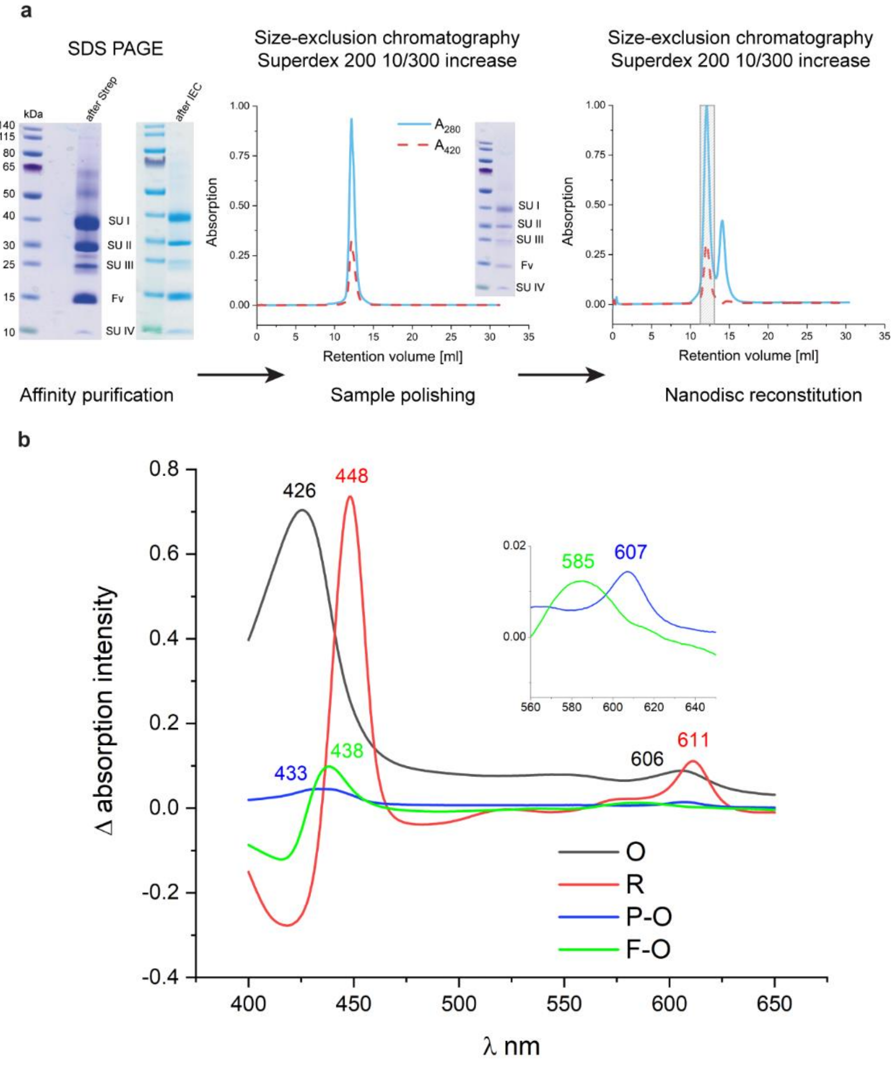

Supplementary Fig. 1 - Sample preparation and uv/visible absorption spectroscopy. (a) The cytochrome *c* oxidase aa_3 from *Paracoccus denitrificans* was purified by Strep-tag affinity chromatography, ion-exchange chromatography and followed by size-exclusion chromatography. The purified and polished protein complex was subsequently reconstituted into lipid nanodiscs (MSP1D1). Empty nanodiscs were separated by a final SEC polishing step. Peak fractions were collected, pooled, concentrated and adjusted to the desired protein concentration for subsequent cryo-EM studies. **(b)** The uv/visible absorption spectra (375 nm - 675 nm) of all intermediate states of interest. For the **P** (blue) and **F** (green) state, the induced-minus-oxidized difference spectra are indicated. Each intermediate was identified by its unique spectroscopic fingerprint

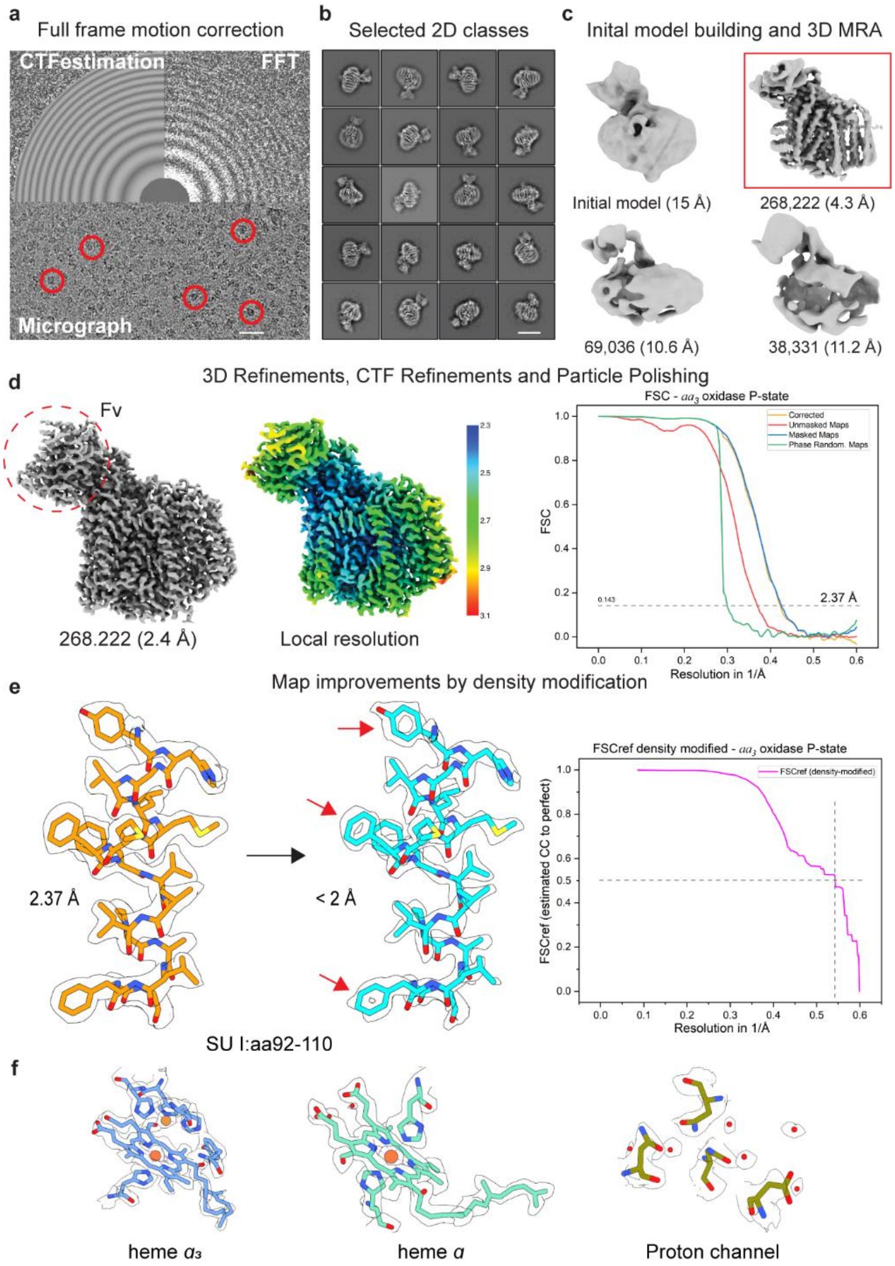
a
Full frame motion correction
CTFestimation
FFT
Micrograph
b
Selected 2D classes
c
Inital model building and 3D MRA
Initial model (15 Å)
268,222 (4.3 Å)
69,036 (10.6 Å)
38,331 (11.2 Å)
d
3D Refinements, CTF Refinements and Particle Polishing
Fv
268.222 (2.4 Å)
Local resolution
FSC - aa3 oxidase P-state
Corrected
Unmasked Maps
Masked Maps
Phase Random. Maps
FSC
0.143
2.37 Å
Resolution in 1/Å
e
Map improvements by density modification
2.37 Å
< 2 Å
SU I:aa92-110
FSCref density modified - aa3 oxidase P-state
FSCref (density-modified)
FSCref (estimated CC to perfect)
Resolution in 1/Å
f
heme a3
heme a
Proton channel

Supplementary Fig. 2 - Data and image processing. Each dataset (**R**, **O**, $\mathbf{P_{CO}}$ and **F-**induced state) was processed in a standardized procedure by using RELION 3.1, here exemplarily shown for the $\mathbf{P_{CO}}$-state. Values for the other datasets are summarized in table S.1. **(a)** Full frame motion correction, CTF estimation (CTFFind4) and automated particle image selection was done in a first step. Scale bars are 30 nm in the micrograph. **(b)** 375,589 particle images from selected 2D classes were picked, merged and further processed. Scale bars are 10 nm. **(c)** Initial model building and 3D multi-reference alignment (MRA) with three classes was performed. Class 1 (268,222 particles), showing clear secondary structural features, was selected. **(d)** Particle images from class 1 were subsequently refined (3D and CTF refinement) and polished, ending up with an average resolution of $FSC_{0.143}$ = 2.37 Å. Excluding the Fv fragment did not improve the resolution but lowered the *B*-factor. The final reconstruction shows differences in local resolution, especially for the catalytic core (SUI) with a local resolution beyond 2.37 Å. **(e)** An extensive density-modification procedure for improving the resolution was applied. Hereby, the density maps from the 2.37 Å dataset in combination with a model and mask file were used, showing a significant improvement in resolution and map quality. Illustrated map densities are filtered to equal contour levels of 1.5. **(f)** Assignment of molecules around the hemes and proton networks was improved and became more discernable by using the density-modification procedure. The corresponding density map is illustrated as a white surface. Illustrated map densities are filtered to equal contour levels of 1.5

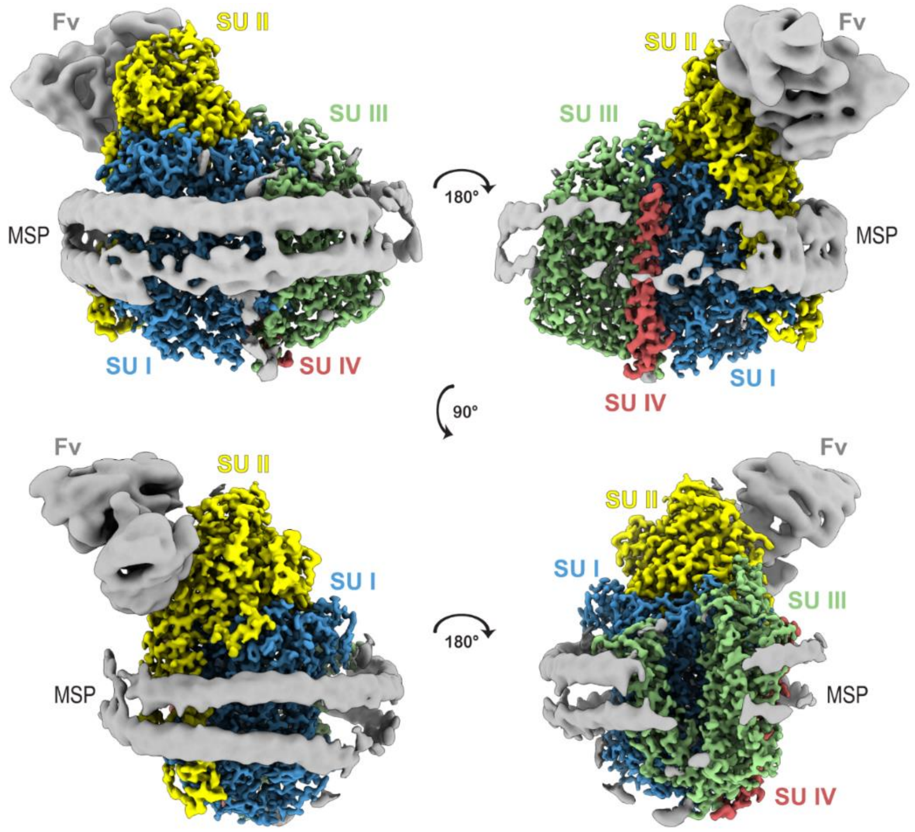

Supplementary Fig. 3 - Cryo-EM density map of the reconstituted *aa*$_3$ oxidase. Surface representation of the fully assembled, reconstituted cytochrome *c* complex (**P**-state dataset) with the Fv fragment attached to the periplasmic surface of SU I. The membrane scaffold protein (MSP) encompasses the membrane protein as a double layer as can be seen. Each subunit (SU I, II, III and IV) is indicated. The illustrated map densities are filtered to equal contour levels of 2.

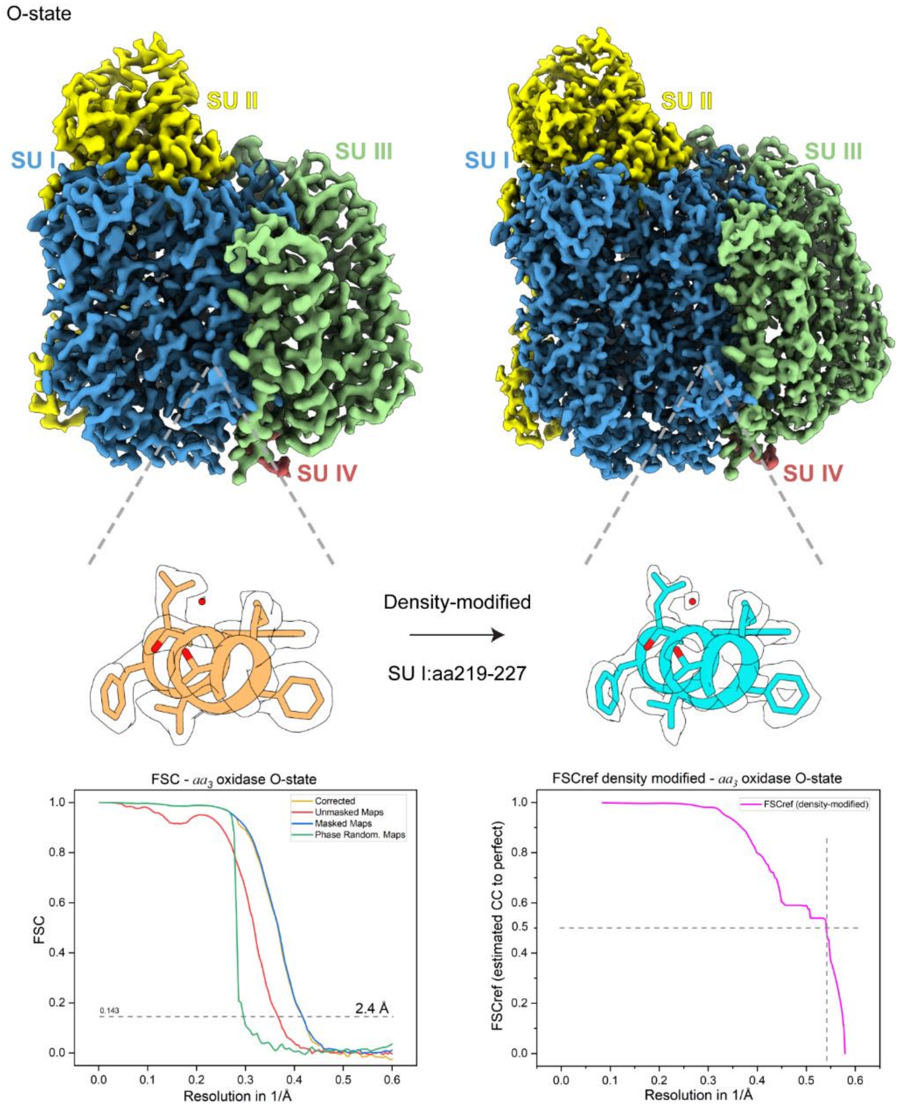

Supplementary Fig. 4 - O-state of the cytochrome *c* oxidase. Distinct resolution improvements of the final 3D reconstruction of C*c*O (**O**-state) were achieved by excluding the signal of the Fv fragment, the nanodisc and using the density-modification method. The corresponding density map is illustrated as a white surface. The illustrated map densities are filtered to equal contour levels of 1.

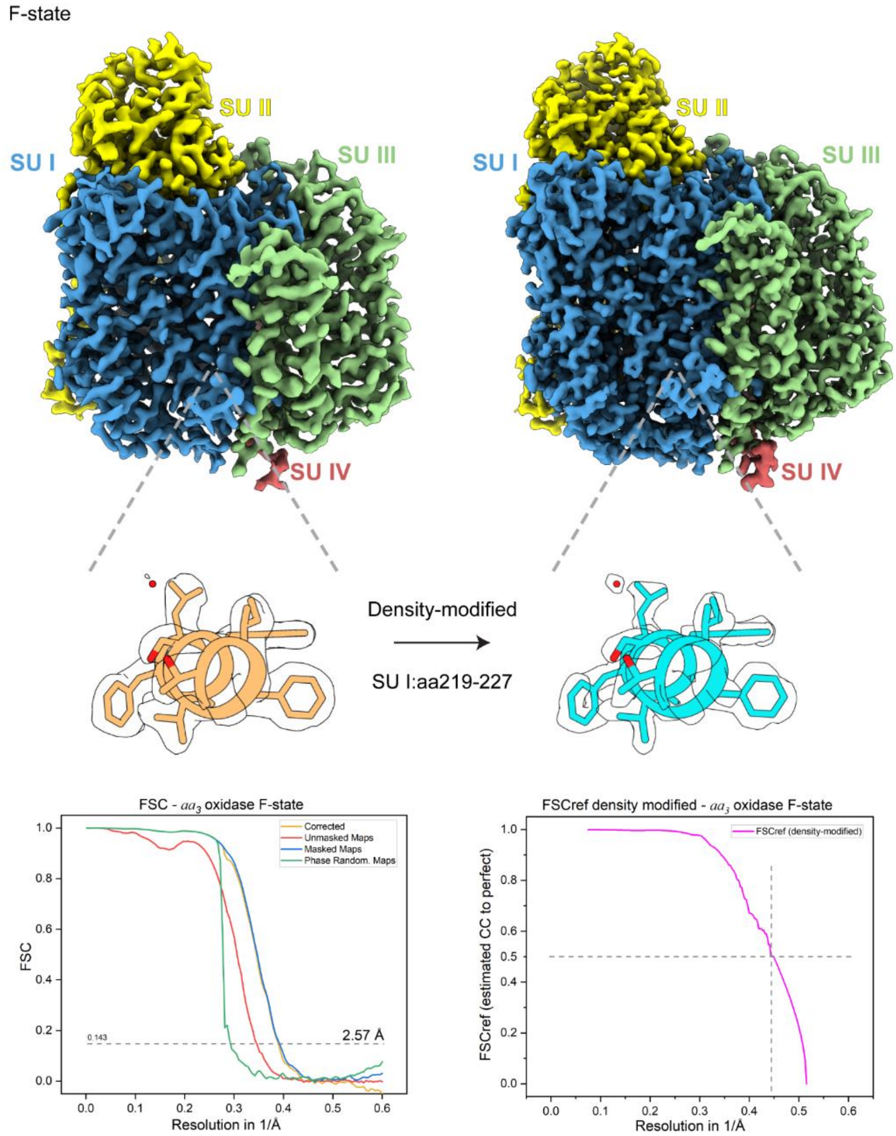

Supplementary Fig. 5 - F-state of the cytochrome *c* oxidase. Distinct resolution improvements of the final 3D reconstruction of C*c*O (**F**-state) were achieved by excluding the signal of the Fv fragment, the nanodisc and using the density-modification method. The corresponding density map is illustrated as a white surface. The illustrated map densities are filtered to equal contour levels of 1.

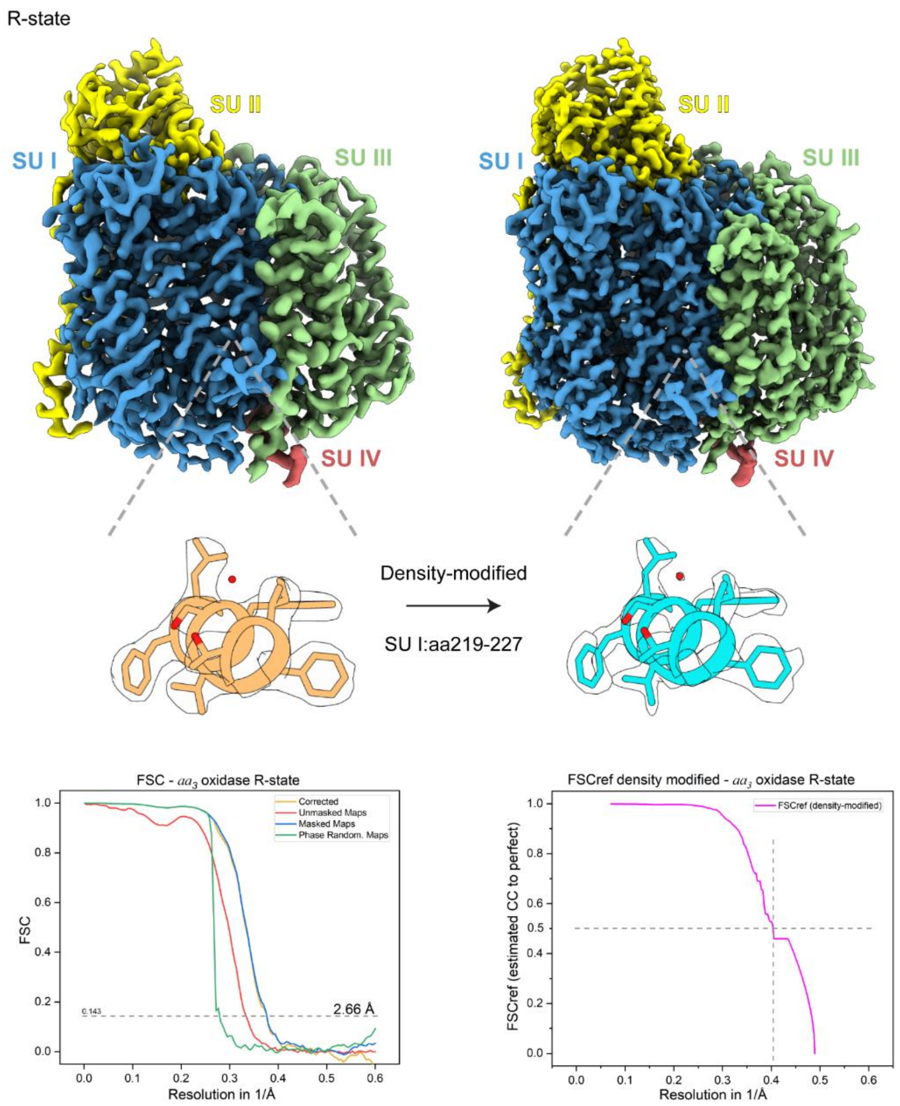

Supplementary Fig. 6 - R-state of the cytochrome *c* oxidase. Distinct resolution improvements of the final 3D reconstruction of C*c*O (**R-**state) were achieved by excluding the signal of the Fv fragment, the nanodisc and using the density-modification method. The corresponding density map is illustrated as a white surface. The illustrated map densities are filtered to equal contour levels of 1.

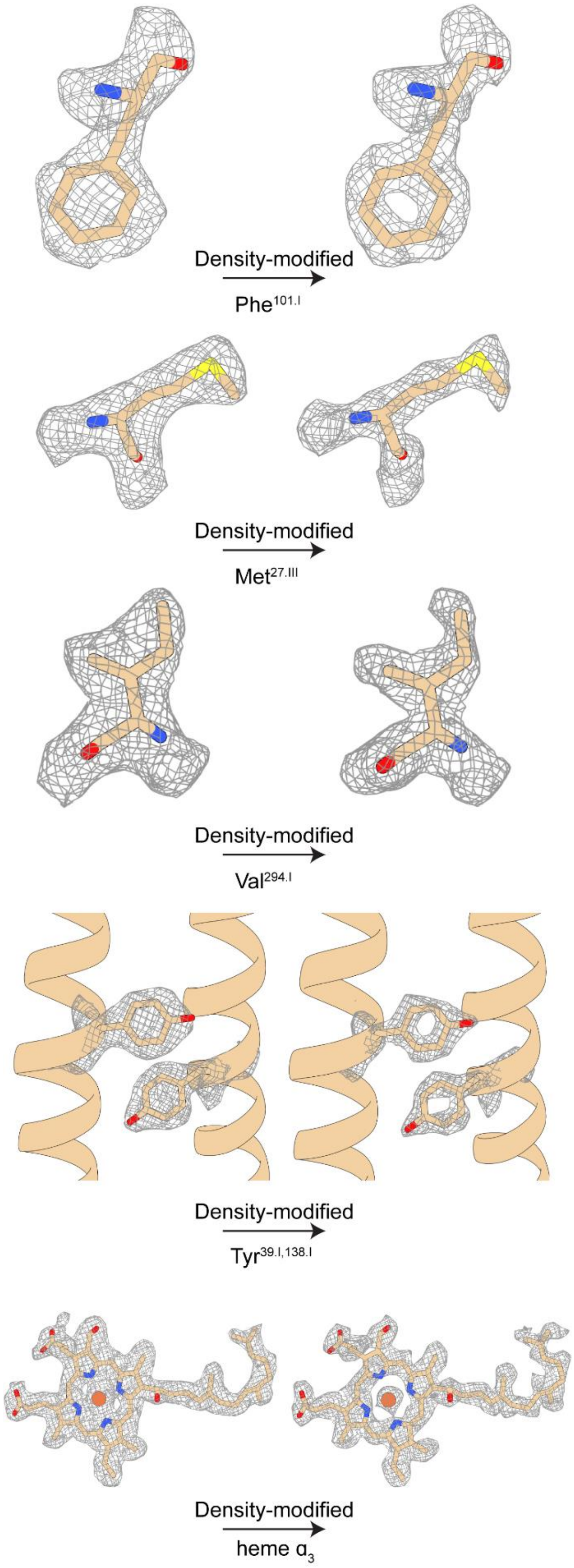

Supplementary Fig. 7 - Resolution improvements. By applying density modification and local masking, overall distinct resolution improvements were achieved. Examples of this are shown. The corresponding density map is illustrated as mesh volumes. The illustrated map densities are filtered to equal contour levels of 1.5.

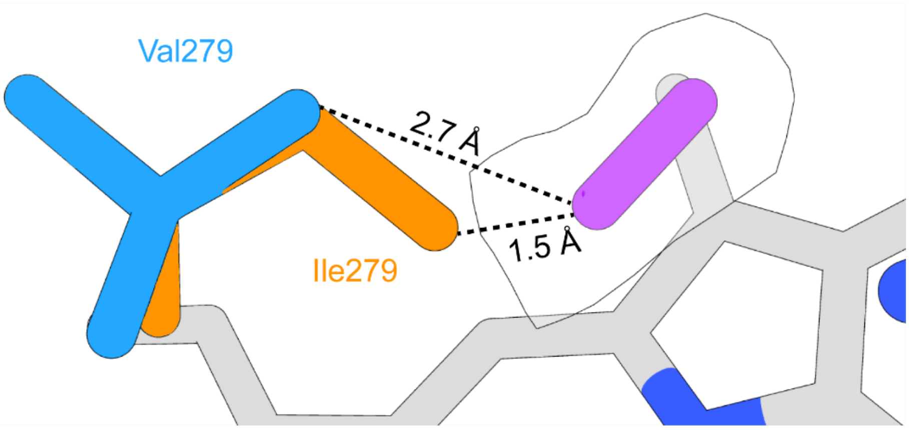

Supplementary Fig. 8 - Replacement of valine to isoleucine. Oxygen uptake at the active site is sterically prevented by the exchange of valine to isoleucine as the longer side chain arm points into the newly discovered oxygen channel which ends near the binuclear center. The molecular oxygen is shown as purple and the corresponding density map is illustrated as a white surface. The illustrated map densities are filtered to equal contour levels of 0.8.

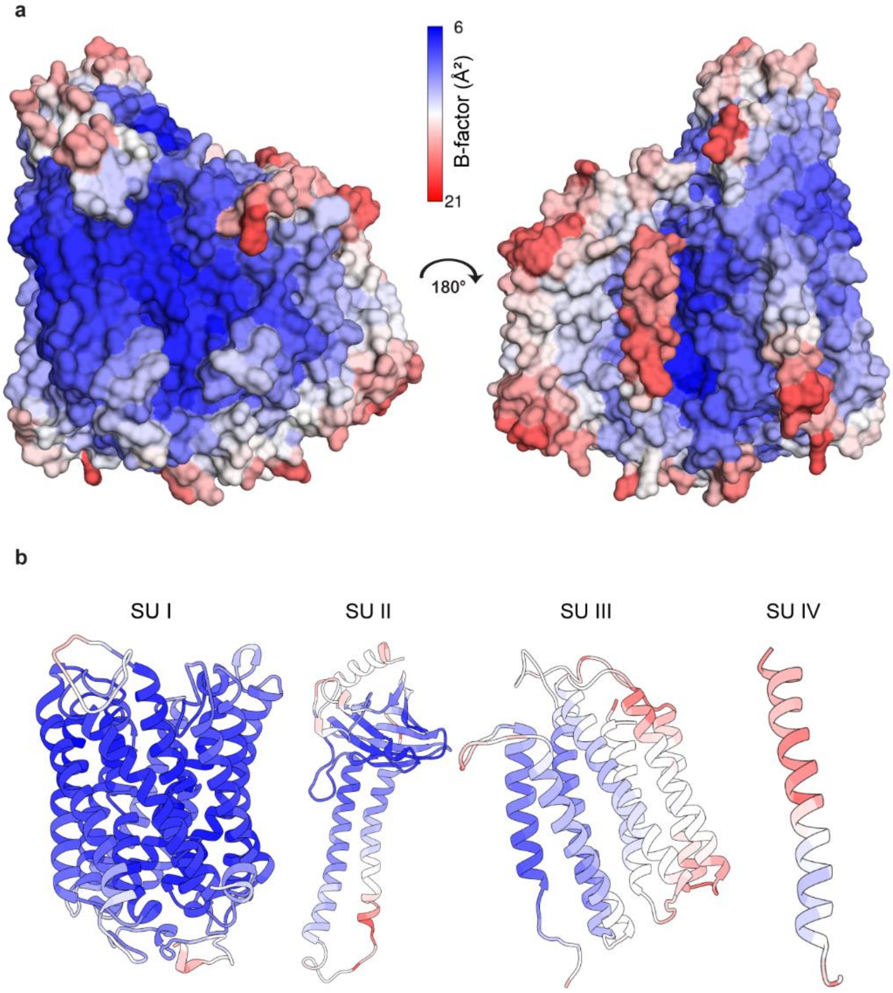

Supplementary Fig. 9 - *B*-factor coloring. (a) Surface representation of the four-subunit cytochrome *c* oxidase colored according to the overall *B*-factor. **(b)** Ribbon representation of each subunit. Higher temperature factors can be seen for subunit III and IV.
