# Supplementary material for: Cryo-EM structures of intermediates suggest an alternative catalytic reaction cycle for cytochrome c oxidase
Source: Nat Commun. 2021 Nov 25;12:6903. doi: 10.1038/s41467-021-27174-y (PMC8617209; doi:10.1038/s41467-021-27174-y)
Supplement: Supplementary file 2 — Reporting Summary [file 41467_2021_27174_MOESM2_ESM.pdf]

nature portfolio

Corresponding author(s): Prof. Dr. Dr. Hartmut Michel

Last updated by author(s): Oct 26, 2021

Reporting Summary

Nature Portfolio wishes to improve the reproducibility of the work that we publish. This form provides structure for consistency and transparency in reporting. For further information on Nature Portfolio policies, see our Editorial Policies and the Editorial Policy Checklist.

Statistics

For all statistical analyses, confirm that the following items are present in the figure legend, table legend, main text, or Methods section.

n/a	Confirmed	
[ ]	[x]	The exact sample size (*n*) for each experimental group/condition, given as a discrete number and unit of measurement
[ ]	[x]	A statement on whether measurements were taken from distinct samples or whether the same sample was measured repeatedly
[x]	[ ]	The statistical test(s) used AND whether they are one- or two-sided<br>*Only common tests should be described solely by name; describe more complex techniques in the Methods section.*
[x]	[ ]	A description of all covariates tested
[x]	[ ]	A description of any assumptions or corrections, such as tests of normality and adjustment for multiple comparisons
[ ]	[x]	A full description of the statistical parameters including central tendency (e.g. means) or other basic estimates (e.g. regression coefficient) AND variation (e.g. standard deviation) or associated estimates of uncertainty (e.g. confidence intervals)
[x]	[ ]	For null hypothesis testing, the test statistic (e.g. *F*, *t*, *r*) with confidence intervals, effect sizes, degrees of freedom and *P* value noted<br>*Give P values as exact values whenever suitable.*
[ ]	[x]	For Bayesian analysis, information on the choice of priors and Markov chain Monte Carlo settings
[x]	[ ]	For hierarchical and complex designs, identification of the appropriate level for tests and full reporting of outcomes
[x]	[ ]	Estimates of effect sizes (e.g. Cohen's *d*, Pearson's *r*), indicating how they were calculated

Our web collection on statistics for biologists contains articles on many of the points above.

Software and code

Policy information about availability of computer code

Data collection	Titan Krios microscope operated at 300 kV and equipped with a BioQuantum energy filter and Falcon 3 camera; Data collection quality was monitored through EPU v. 2.9.
Data analysis	Chimera v. 1.15, ChimeraX v.1.1, MotionCor2, CTFFind-4.1, RELION-3.1; COOT v. 0.8.9; Phenix v. 1.14; MolProbity v. 4.5; MOLE 2.5, EPU v. 2.9;

For manuscripts utilizing custom algorithms or software that are central to the research but not yet described in published literature, software must be made available to editors and reviewers. We strongly encourage code deposition in a community repository (e.g. GitHub). See the Nature Portfolio guidelines for submitting code & software for further information.

Data

Policy information about availability of data

All manuscripts must include a data availability statement. This statement should provide the following information, where applicable:

- Accession codes, unique identifiers, or web links for publicly available datasets
- A description of any restrictions on data availability
- For clinical datasets or third party data, please ensure that the statement adheres to our policy

The Cryo-EM maps of the cytochrome c oxidase from P. denitrificans are deposited at the Electron Microscopy Data Bank (https://www.ebi.ac.uk/emdb) under accession numbers EMD-11921, EMD-11922, EMD-11924 and EMD-11925. Each model of the cytochrome c oxidase structure was submitted to the Protein Data Base (https://www.wwpdb.org/) with following accession numbers: 7ATN , 7ATE, 7AU3 and 7AU6 . 1QLE was used as a template.

Field-specific reporting

Please select the one below that is the best fit for your research. If you are not sure, read the appropriate sections before making your selection.

- [x] Life sciences
- [ ] Behavioural & social sciences
- [ ] Ecological, evolutionary & environmental sciences

For a reference copy of the document with all sections, see nature.com/documents/nr-reporting-summary-flat.pdf

Life sciences study design

All studies must disclose on these points even when the disclosure is negative.

Sample size	Sample sizes (number of collected micrographs) of respective cryo-EM datasets were chosen based on instrument availability and applied dose rate. Datasets of around 5000 micrographs for a respective sample ensured a sufficient number of particles to achieve resolutions < 2.6 Å. The smallest collected dataset contained 4487 micrographs (P-state) while the largest dataset contained 5835 micrographs (R-state).
Data exclusions	No data were excluded
Replication	Simple particle cryo-EM is based on averaging protein particles of nearly identical orientation within a vitreous layer of ice. Therefore, replication is not per se required to ensure statistical robustness of structural data. In case of this work, we have determined four individual structures of cytochrome c oxidase under different sample conditions. In all of these structures, the general architecture of cytochrome c oxidase remained identical. Hence these data can be considered as biological replicates of the presented structural data.
Randomization	Generally, no randomization was required for the experimental design of this study. However, it is to note that particles are randomized during data processing steps in Relion-3.1 (randomization during 2D classification, randomized half sets during Refine3D). Randomized half sets of particles are used in final reconstruction steps in order to determined gold-standard Fourier shell correlations based on the 0.143 level.
Blinding	This study was performed on a single enzyme sample. The experimental design did not require for blinding approaches in order to ensure robustness of obtained structural and biochemical data. Furthermore, SPA cryo-EM includes averaging of individual particles and weighting algorithms resulting in electron densities that are inheretly unbiased. Randomized half maps during gold-standard FSC calculation moreover ensure adequate resolution estimations.

Reporting for specific materials, systems and methods

We require information from authors about some types of materials, experimental systems and methods used in many studies. Here, indicate whether each material, system or method listed is relevant to your study. If you are not sure if a list item applies to your research, read the appropriate section before selecting a response.

Materials & experimental systems

n/a	Involved in the study	
☐	☒	Antibodies
☒	☐	Eukaryotic cell lines
☒	☐	Palaeontology and archaeology
☒	☐	Animals and other organisms
☒	☐	Human research participants
☒	☐	Clinical data
☒	☐	Dual use research of concern

Methods

n/a	Involved in the study	
☒	☐	ChIP-seq
☒	☐	Flow cytometry
☒	☐	MRI-based neuroimaging

Antibodies

Antibodies used	Non-clinical bioengineered antibody fragment (Fv) was used as a tool for isolating and purifying the cytochrome c oxidase from P. dentrificans. This work was published by Kleymann et al. (1995).
Validation	Purification of cytochrome c oxidase from P. denitrificans is possible exclusively with the Fv fragment because it contains a Strep binding site and binds specifically to the second subunit of cytochrome c oxidase.
